# HIV-1 Amplifies IL-8 Response Of Human Stellate Cells To Gram-Positive Microbial Products Via H4K5 Histone Acetylation

**DOI:** 10.1101/2025.05.12.653460

**Published:** 2025-05-15

**Authors:** Lumin Zhang, Rabab O. Ali, Naichaya Chamroonkul, Parissa Tabrizian, Myron Schwartz, Ganesh Gunasekaran, Thomas Schiano, Maria Isabel Fiel, Steven Ward, Meena B. Bansal

## Abstract

**Background:**

Patients living with Human Immunodeficiency Virus (PLWH) develop accelerated liver fibrosis, but the exact mechanism remains unknown. Hepatic stellate cell (HSC) activation is the cornerstone for liver fibrosis and influenced by multiple factors, such as viral infection, hepatocellular injury, chronic immune activation, gut mucosal barrier dysfunction, and microbial translocation. Unlike gram-negative bacterial products, the impact of gram-positive microbial products in Human Immunodeficiency Virus 1 (HIV-1) associated liver inflammation and fibrosis remains poorly understood. In this study, we investigated the effect of lipoteichoic acid (LTA), a major gram-positive bacterial component, on HSCs in the context of HIV-1 infection.

**Methods:**

Human HSCs were isolated from the livers of both non-HIV and HIV-infected patients undergoing hepatic resection. The inflammatory responses of HSCs to LTA stimulation were measured by ELISA before or after HIV-1BaL ex vivo expision. Western blotting, ChIP-qPCR and RNA-seq were used to reveal the mechanisms contributing to the IL-8 response to LTA stimulation and HIV-1BaL exposure in HSCs.

**Results:**

While LTA stimulation modestly increased IL-8 production in HSCs, this effect was significantly amplified in HIV-1-infected HSCs. Elevated IL-8 levels were detected in HIV-1+ liver sections, and IL-8 treatment of cultured HSCs significantly increased α-SMA and COL1A1 expression, supporting IL-8 as a key inflammatory driver of liver fibrosis in HIV-1 ^+^ individuals. Transcriptomic analyses implicated histone acetylation as a regulator of the enhanced IL-8 response of HSCs to LTA in HIV-1 infection. Consistent with this, treatment with Trichostatin A (TSA), a histone deacetylase (HDAC) inhibitor, further increased the IL-8 response in HIV-1-infected HSCs. ChIP-qPCR confirmed that histone H4K5 acetylation facilitated IL-8 promoter transactivation, rendering HIV-1-infected cells more responsive to LTA.

**Conclusions:**

HIV-1 infection sensitizes HSCs to LTA stimulation, leading to an amplified IL-8 response mediated through histone acetylation that may accelerate liver fibrosis progression in PLWH. As microbial translocation persists despite effective antiretroviral therapy, these findings underscore the need for targeted strategies to mitigate liver fibrosis in HIV-1 infected HIV-1 ^+^ individuals.

## Full Text Availability

The license terms selected by the author(s) for this preprint version do not permit archiving in PMC. The full text is available from the preprint server.

